# Medicine

**Published:** 1907-09-07

**Authors:** 


### HANDBOOKS FOR THE SENIOR STUDENT AND THE PRACTITIONER.

### MEDICINE.

"Osler's System of Medicine." Edited by Osler and McCrae. (7 vols.). 24s. each volume. Oxford Medical Publications, 20 Warwick Square, E.C. (Only Vols. I. and II. have as yet appeared, and judging from them the system is likely to prove one of the best which it is possible to obtain.)

"Allbutt's System of Medicine." Edited by Professor Clifford Allbutt. 8 vols., 25s. each. Macmillan and Co. (A new edition is in course of publication, of which the first three volumes have already appeared. Allbutt is deservedly ranked as one of the finest works on medicine, and is specially valuable for practitioners because of the great amount of space given to consideration of treatment and pathology.)

"Allchin's Manual of Medicine." 5 vols., 7s. 6d. and 10s. 6d. each. Macmillan and Co. (Shorter but very useful, and particularly good on diseases of special systems.)

"Roberts' Medicine." 26s. 2 vols. H. K. Lewis, Gower Street. (Fully up to date and practical; well written.)

"Gibson's Medicine." 2 vols. 25s. net. Young J. Pentland, Teviot Place, Edinburgh.

"Osler's Medicine." 1 vol. Young J. Pentland. 24s.

"Taylor's Medicine." Churchill, 7 Great Marlborough Street, 15s. net.

"Savill's Medicine." 2 vols., 12s. 6d. each. Churchill.

"Fagge and Pye Smith's Medicine." 2 vols. 42s. net. Churchill.

"Charteris's Medicine." Churchill. 10s. net.

"Bain's Medicine." Longmans, Green and Co. 25s.

"Bury's Clinical Medicine." 1 vol. 16s. Charles Griffin and Co., Exeter Street, London.

"Monro's Medicine." Baillière, Tindall, and Cox. 1 vol. 15s.

"Wheeler's Medicine." 8s.

"Hare's Medicine." 2 vols. 21s. Henry Kimpton.

